# Therapeutic Effects of Water Soluble Danshen Extracts on Atherosclerosis

**DOI:** 10.1155/2013/623639

**Published:** 2013-01-16

**Authors:** Yoon Hee Cho, Cheol Ryong Ku, Zhen-Yu Hong, Ji Hoe Heo, Eun Hee Kim, Dong Hoon Choi, Dongkyu Kim, Ae-Jung Kim, Cheol Soon Lee, Mankil Jung, Hyun Chul Lee, MiRan Seo, Eun Jig Lee

**Affiliations:** ^1^Severance Hospital Integrative Research Institute for Cerebral & Cardiovascular Diseases, Severance Hospital, Seoul 120-752, Republic of Korea; ^2^Division of Endocrinology, Department of Internal Medicine, Yonsei University College of Medicine, Seoul 120-752, Republic of Korea; ^3^Division of Neurology, Yonsei University College of Medicine, Seoul 120-752, Republic of Korea; ^4^Division of Cardiology, Department of Internal Medicine, Yonsei University College of Medicine, Seoul 120-752, Republic of Korea; ^5^eGene, Inc., Lee Gil Ya Cancer and Diabetes Institute, Republic of Korea; ^6^Department of Chemistry, Yonsei University, Seoul 120-752, Republic of Korea

## Abstract

Danshen is a traditional Chinese medicine with many beneficial effects on cardiovascular diseases. The aim of this study was to evaluate the mechanisms responsible for the antiatherogenic effect of water soluble Danshen extracts (DEs). Rat vascular smooth muscle cells (VSMCs) and human umbilical vein endothelial cells (HUVECs) were treated with DE. To evaluate the effects of DE *in vivo*, carotid balloon injury and tail vein thrombosis were induced in Sprague-Dawley (SD) rats and iliac artery stent was induced in New Zealand white rabbits. The inhibitory action of DE on platelet aggregation was confirmed with an impedance aggregometer. DE inhibited the production of reactive oxygen species, and the migration and proliferation of platelet-derived growth factor-BB stimulated VSMCs. Furthermore, DE prevented inflammation and apoptosis in HUVECs. Both effects of DE were reconfirmed in both rat models. DE treatment attenuated platelet aggregation in both *in vivo* and *ex vivo* conditions. Pretreatment with DE prevented tail vein thrombosis, which is normally induced by **κ**-carrageenan injection. Lastly, DE-treated rabbits showed decreased in-stent restenosis of stented iliac arteries. These results suggest that water soluble DE modulates key atherogenic events in VSMCs, endothelial cells, and platelets in both *in vitro* and *in vivo* conditions.

## 1. Introduction

Danshen (*Salvia miltiorrhiza*) has been used for the treatment of cardiovascular and cerebrovascular diseases [1, 2]. Specific clinical uses include angina pectoris, hypercholesterolemia, and acute ischemic stroke. This traditional medicine is a popular drug in China, where it is used either on its own or mixed with other herbs [3]. Even in the United States, Danshen has been widely used in recent years [4, 5]. Specifically, the Fufang Danshen Dripping Pill has cleared American Phase II clinical trials in patients with chronic stable angina pectoris (http://clinicaltrials.gov/, no. NCT00797953). The beneficial effects of Danshen on cardiovascular diseases arise from its ability to prevent atherosclerosis. Danshen, as a therapeutic agent for cardiovascular diseases, contributes to improved microcirculation, vasodilation, anticoagulation, and anti-inflammation [6, 7]. In addition to its effects on cardiovascular diseases, some studies have shown that Danshen may be useful in a diverse range of diseases including liver fibrosis, chronic renal failure, and acute pancreatitis [2].

Recently, several chemical compounds belonging to two specific classes were isolated from Danshen; caffeic acid-derived phenolic acids and tanshinones belonging to the diterpene quinine family [8]. According to the solubility, these compounds could be simply classified as either water-soluble agents or lipid-soluble agents. Most studies have reported that the lipid-soluble components of Danshen extracts (DE), such as tanshinone IIA, have specific effects on cardiovascular disease [9].

Although the beneficial effects of Danshen on cardiovascular diseases have been reported in other studies, the underlying mechanisms of DE as well as the role of its water-soluble compounds have not yet been evaluated. With respect to the pharmacological and therapeutic profiles of Danshen in the vascular system, the aim of the present study was to evaluate the mechanisms involved in the antiatherogenic effects of DE, which contains an abundance of water-soluble compounds.

## 2. Materials and Methods

This study conforms to the Guide for the Care and Use of Laboratory Animals published by the US National Institutes of Health (NIH Publication, 8th Edition, 2011). Furthermore, approval was granted by the Institutional Animal Care and Use Committee of Yonsei University Health System (permit number 2010-0187). All animal studies were performed in facilities approved by the Association for Assessment and Accreditation of Laboratory Animal Care. In all animal models, animals were anesthetized with an intraperitoneal injection of Ketamine-Xylazine (100 mg : 10 mg per kg of body weight) once every procedure. The adequacy of anesthesia in Sprague-Dawley (SD) was assessed by absence of reflexes prior to rapid cervical dislocation, and that in New Zealand white rabbits was confirmed by monitoring respiratory rate and ECG.

### 2.1. Production of Danshen Extracts

Dried and powdered roots of *Salvia miltiorrhiza* (200 g) were boiled with water (1600 mL) for 2 hours. The extracted solution was then filtered and solid deposits were removed. The extracted solution was then concentrated by heating with a rotary evaporator. Next, identical volumes of extracted solution and butanol were mixed in a separatory funnel. The upper solution of the mixture was then collected and concentrated with a rotary evaporator. The components of the collected DE were evaluated via ultra performance liquid chromatography-ultraviolet (UPLC-UV). Powdered DE was dissolved in distilled water containing 1% dimethyl sulfoxide (DMSO) (Sigma-Aldrich Co., MO, USA) and diluted to final experimental concentrations.

### 2.2. Primary Cell Isolation and Culture

Rat vascular smooth muscle cells (VSMCs) were isolated from the thoracic aorta of SD rats weighing between 200 to 250 g (Orient-Charles River Technology, Seoul, Korea), as described previously [10]. VSMCs between passages four and five were used in this study. Human umbilical vein endothelial cells (HUVECs) (Invitrogen Life Technology, CA, USA) were grown in endothelial cell growth medium-2 supplemented with 2% fetal bovine serum (FBS), 0.04% hydrocortisone, 0.4% human epidermal growth factor (hEGF)-B, 0.1% vascular endothelial growth factor (VEGF), 0.1% R3-insulin like growth factor (IGF)-1, 0.1% ascorbic acid, 0.1% hEGF, 0.1% GA-1000, and 0.1% heparin (Lonza, Basel, Switzerland) and synchronized by serum deprivation (0.1% FBS) for 2 h. HUVECs between passages 8 and 10 were used in this study. After synchronization, VSMCs and HUVECs were treated with DE for 24 h prior to stimulation with platelet-derived growth factor (PDGF)-BB (R&D Systems, MN, USA) and tumor necrosis factor (TNF)-*α* (Millipore, MA, USA). PDGF-BB (20 ng/mL) and TNF-*α* (10 ng/mL) were applied to both VSMCs and HUVECs for 24 h. 

### 2.3. Cell Proliferation Analysis, Wound Healing Assay, and Reactive Oxygen Species Assay

Cell proliferation was assessed using a modified 3-[4,5-dimethyl-2-thiazolyl]-2,5-diphenyl-2 tetrazolium bromide (MTT) (Sigma-Aldrich Co.) assay following a standard protocol [11]. Wound healing assays were performed using 6-well plates. When cells reached 90% confluence, synchronized cells were pretreated with DE (100 *μ*g/mL) in serum-free medium for 24 h. After 24 h of PDGF-BB (20 ng/mL) stimulation, a single wound was created in the center of the cell monolayers by gentle removal of the attached cells with a sterile plastic pipette tip. After 24 h of incubation, the cells that migrated into the wounded area or protruded from the border of the wound were visualized and photographed under an inverted microscope. The effect of DE on intracellular reactive oxygen species (ROS) levels was examined as described previously [12]. Briefly, at 24 after PDGF (20 ng/mL) following DE (100 *μ*g/mL) treatment, cells were incubated for 30 min at 37°C with CM-H_2_DCF-DA (Molecular Probes Inc., OR, USA).

### 2.4. Propidium Iodide Staining for Cell Cycle Analysis

At 24 after PDGF (20 ng/mL) treatment following DE (100 *μ*g/mL) pretreatment, cells were harvested and washed with PBS, followed by fixation with 70% ethanol overnight at −20°C. After washing with 3% BSA/PBS twice, the cells were resuspended in PBS containing 50 *μ*g mL propidium iodide and 10 *μ*g mL RNase A for 30 min at room temperature. Samples were analysed for DNA content by flow cytometry. The cell-cycle phases were analysed using CELLQuest software (Becton Dickinson, San Jose, CA, USA).

### 2.5. Immunoblotting

Cell lysates were subsequently prepared and subjected to western blot analysis. Membranes were then immunoblotted with primary antibodies for phosphor-Akt (Ser473) (Cell Signaling), nonmuscle heavy chain II-A (Abcam, MA, USA), manganese superoxide dismutase (MnSOD) (Abcam), lamin A (Santa Cruz, CA, USA), JNK (Cell Signaling Technologies, MA, USA), p65 (Cell Signaling Technologies), and hemoxygenase-1 (HO-1) (Santa Cruz). Peroxidase-conjugated anti-rabbit or anti-mouse antibodies were used as secondary antibodies (Thermo Fisher Scientific). Isolation of nuclear fractions was carried out by using a commercial kit (Cayman Chemical Item Number 10009277). 

### 2.6. Rat Model for Carotid Artery Balloon Injury

The *in vivo* therapeutic effect of DE on neointimal hyperplasia was investigated with a balloon injury animal model as previously described [13]. Male Sprague-Dawley rats (250–300 g; ORIENT-Charles River Technology) were separated into three groups, including vehicle treatment (*n* = 7), DE (10 mg/kg) treatment (*n* = 7), and DE (50 mg/kg) treatment (*n* = 7). DE and vehicle were administered via oral sonde daily beginning two weeks before until four weeks after induction of balloon injury. To evaluate the effect of DE pretreatment on neointimal hyperplasia, SD rats were classified into three groups in separate studies, including vehicle treatment (*n* = 9), DE treatment for four weeks after balloon injury (*n* = 9), and DE pretreatment for two weeks followed by DE treatment for four weeks after balloon injury (*n* = 9). Vehicle or DE (10 mg/kg/d) was administered daily. Four weeks after balloon injury, the morphometric analysis of common carotid arteries was performed by staining with hematoxylin and eosin. Images of each carotid artery section were analyzed by computerized morphometry (Scion Image Software), and luminal, intimal, and medial areas were then calculated for each arterial cross section. Neointimal formation was expressed as a percent computed as follows: [intima/(media + intima)] × 100 (%).

### 2.7. Rat Model for Tail Vein Thrombosis

To evaluate the effect of DE on thrombosis formation, thrombosis was induced via the tail veins of male SD rats (250–300 g) (Orient-Charles River Technology) classified into three groups, namely, vehicle (normal saline, *n* = 6) or DE pretreatment for two weeks before the induction of tail vein thrombosis (10, 50 mg/kg, each *n* = 6). DE was administered daily through oral gavage. Bleeding time was checked after pretreatment for two weeks as described previously [14]. Tail vein thrombosis was achieved with a modified Beckmeier's model as previously described [15]. The length of the thrombosis was evaluated 6, 24, 48, 96, and 168 hours after *κ*-carrageenan injection. Heparin (200 U (mL/Kg)^−1^) was injected i.p. as a positive control for antithrombotic agents 10 minutes after *κ*-carrageenan administration. 

### 2.8. Inhibitory Action on Rat Platelet Aggregation

Platelet aggregation was evaluated using an impedance aggregometer (Chronolog model 700, Chronolog Corporation, Havertown, PA, USA) after *ex vivo* and *in vivo* treatment of DE. For *ex vivo* studies, whole blood (9 mL) was obtained from male SD rats (Orient-Charles River Technology) weighing between 250–300 g. Whole blood was collected in plastic syringes containing heparin (1.5%) (JW Pharmaceuticals, Seoul, Korea) to avoid premature aggregation. Single-use cuvettes containing a silicon-coated stirrer (Chronolog Corporation) (1200 rpm) were filled with 500 *μ*L physiological saline and 500 *μ*L prepared whole blood. After incubating for 15 minutes at 37°C, the aggregation of platelets in whole blood was initiated by adding ADP (20 *μ*M) (Sigma-Aldrich Co., MO, USA) as a stimulating agonist. Pretreatment of DE (0.2 mg/mL, 2.5 mg/mL, and 5.0 mg/mL) was performed 10 minutes prior to the initiation of aggregation. For *in vivo* studies, male SD rats (Orient-Charles River Technology) weighing between 200 to 250 g were classified into three groups, namely, DE (10 mg/kg and 50 mg/kg) and vehicle treatments. In the DE treatment group (each group *n* = 4), DE was administered daily via oral sonde for two weeks. After treatment with DE or normal saline, whole blood was obtained and analyzed with an impedance aggregometer. 

### 2.9. Rabbit Iliac Artery Stent Model

The therapeutic effect of DE on thrombosis was investigated with an iliac artery stent insertion rabbit model. Briefly, the iliac artery stent was inserted as previously described [16]. Male New Zealand white rabbits (*n* = 3) (Orient-Charles River Technology) weighing 3.5 to 4.0 kg were classified into two groups, namely, the normal saline group (*n* = 1) and DE group (*n* = 2). Each agent was injected subcutaneously twice a day from two weeks before to four weeks after the procedure. DE was dissolved in normal saline containing 1% DMSO and injected with a single 20 mg/kg dose. Aspirin was administered orally once a day at a dose of 100 mg/animal to all rabbits, beginning two days before the procedure and continued until sacrifice. Four weeks after the procedure, stented arteries were evaluated with intravascular optical coherence tomography (OCT). Animals were subsequently sacrificed and the stented segments of both iliac arteries were harvested. Using OCT and harvested arteries, the size of in-stent restenosis was calculated. 

### 2.10. Statistical Analyses

Data were analyzed using Mann-Whitney tests. All statistical analyses were performed using SPSS (ver. 13.0 for Windows; SPSS, Inc., Chicago, IL, USA). All statistical tests were two-tailed, and *P* values <0.05 were considered significant.

## 3. Results

### 3.1. Danshen Extract Contains an Abundance of Water-Soluble Components

Twelve compounds were utilized as reference agents in UPLC-UV [17]. They included salvianic acid (danshensu), dihydroxybenzoic acid (protocatechuic acid), dihydroxybenzaldehyde (protocatechuic aldehyde), caffeic acid, rosmarinic acid, lithospermic acid, salvianolic acid B, salvianolic acid A, dihydrotanshinone I, cryptotanshinone, tanshinone I, and tanshinone IIA. Most of the compounds in DE were water soluble, whereas the number of solely liposoluble compounds was minimal (Table 1).

### 3.2. Danshen Extract Has Antioxidant, Antimigratory and Antiproliferative Effects on PDGF-BB Stimulated VSMCs

**Figure 1:**
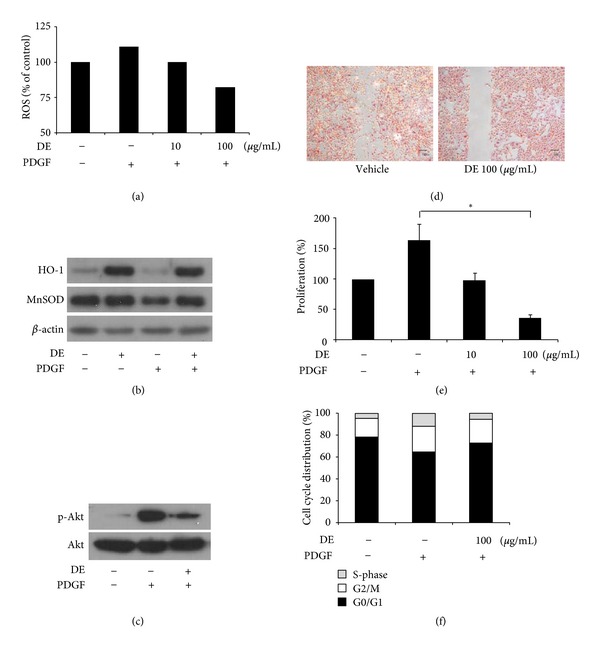
The antioxidant, antimigratory, and antiproliferative effects of Danshen extract on PDGF-BB-treated vascular smooth muscle cells. Platelet-derived growth factor (PDGF)-BB (20 ng/mL) was treated for 24 h after Danshen extracts (DE) treatment at each concentration for 24 h. (a) Fluorescence activated cell sorting for PDGF-BB-induced intracellular reactive oxygen species in vascular smooth muscle cells (VSMCs). (b) Western blotting for manganese superoxide dismutase and hemoxygenase-1. DE (100 *μ*g/mL) treated for 24 h before PDGF treatment. (c) Western blotting for proteins on Akt signaling pathway after treatment DE (100 *μ*g/mL) for 24 h. (d) The wound-healing experiment with or without DE (100 *μ*g/mL, 24 h). (e) Proliferation assessed by the MTT cell proliferation assay. Relative proliferation activities were expressed using untreated control cells as a standard. Results are expressed as the mean ± standard error; *P < 0.05 versus vehicle treatment after PDGF-BB induction. (f) The effects of DE on cell cycle progression. DE: Danshen extract.

The effects of DE on ROS generation, migration, and proliferation of VSMCs were evaluated by PDGF-BB induction. According to the CM-H_2_DCF-DA emission results from FACS, DE most significantly decreased the amount of ROS at a concentration of 100 *μ*g/mL (Figure 1(a)). Further *in vitro* studies were conducted with a 100 *μ*g/mL concentration of DE, the levels of which increased MnSOD and HO-1 expression. Both are important molecules for the inhibition of VSMC proliferation and ROS production. The expression of HO-1 was increased by DE even after PDGF-BB treatment. On MnSOD, DE treatment prevented the decreased expression induced by PDGF-BB (Figure 1(b)). To evaluate changes in PDGF signaling after DE treatment, the expression of PI3K/Akt was analyzed. Although PDGF-BB (20 ng/mL, 24 h) stimulated the phosphorylation of Akt, this effect was blocked by DE treatment with a dose of 100 *μ*g/mL (Figure 1(c)). 

According to migration studies with VSMCs, PDGF (20 ng/mL, 24 h) treatment resulted in wound-induced migration (Figure 1(d)). However, DE treatment significantly prevented PDGF induced wound migration. For MTT assays, quiescent cells were treated with DE (100 *μ*g/mL for 24 h) in the absence of PDGF-BB, and then stimulated with PDGF-BB (20 ng/mL, 24 h). Proliferation of VSMCs treated with PDGF-BB was 53% higher compared to that in PDGF-BB (−) control cells. DE inhibited PDGF-BB-induced VSMC proliferation in a dose-dependent manner, up to 65.7% inhibition at 100 *μ*g/mL of DE (Figure 1(e)). According to the cell cycle analysis, DE blocked the induction of S-phase entry, which normally occurs in PDGF-stimulated VSMCs (Figure 1(f)). 

### 3.3. Danshen Extract Exerts Anti-Inflammatory and Antiapoptotic Effects on HUVECs

**Figure 2:**
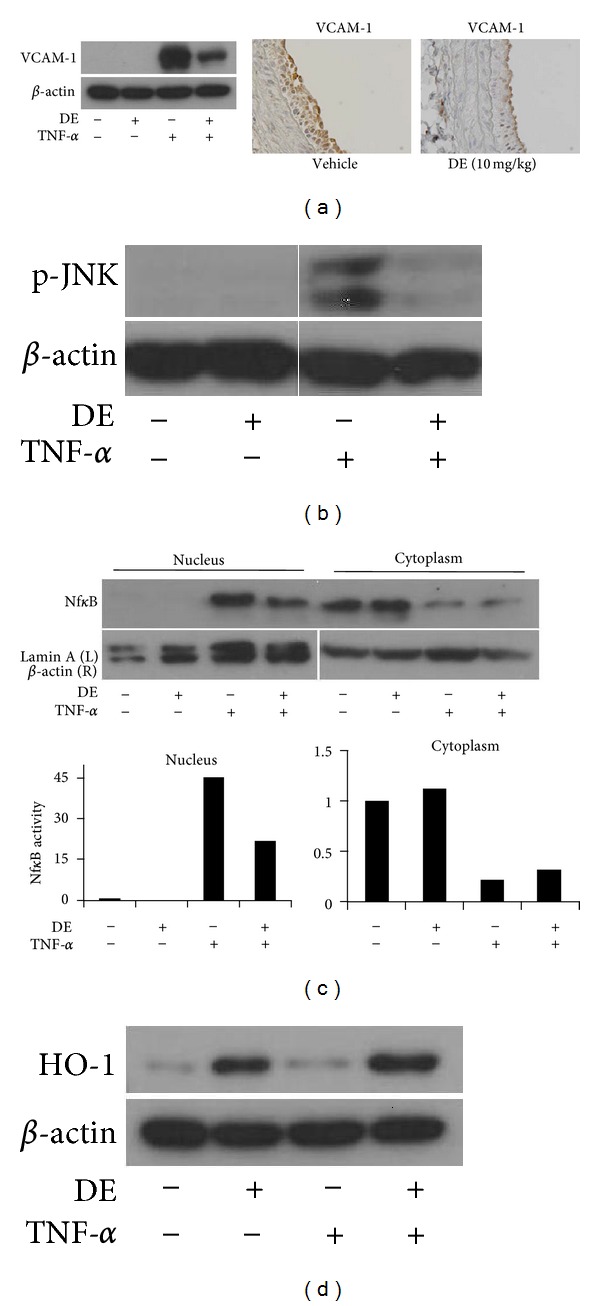
Anti-inflammatory and antiapoptotic effects of Danshen extract on human umbilical vein endothelial cells. (a) Western blotting (left panel) and immunohistochemical staining (right panel) for VCAM-1. Human umbilical vein endothelial cells (HUVECs) were pretreated with Danshen extract (DE) (100 *μ*g/mL) 24 h prior to exposure with TNF-*α* (10 ng/mL) for 24 h. Representative immunohistologic sections (200X) of VCAM-1 in balloon-injured rat carotid arteries were shown. (b)-(c) Western blotting for phosphorylated JNK and NF-*κ*B p65. HUVECs were treated with TNF-*α* (100 ng/mL) for 15 m with or without pretreatment with DE (100 *μ*g/mL) for 24 h. (d) Western blotting for hemoxygenase-1 in both HUVECs with or without treatment of TNF-*α* (100 ng/mL) for 15 min. DE: Danshen extract.

To determine the effects of DE on endothelial cells, HUVECs with or without DE pretreatment for 24 h were incubated with TNF-*α* (10 ng/mL) for 24 hours. TNF-*α* alone increased the expression of VCAM-1 in HUVECs, whereas DE pretreatment significantly inhibited TNF-*α* induced VCAM-1 expression (Figure 2(a)). The effect of DE on VCAM-1 expression in the HUVECs correlated with that of balloon injured rat arteries. Although endothelium exhibited a significant increase in VCAM-1 expression after balloon injury, the expression of VCAM-1 in those of rats who had been given DE was significantly decreased (Figure 2(a)).

To determine the effect of DE on upstream signaling of VCAM-1, changes to the mitogen-activated protein kinase (MAPK) and nuclear factor (NF)-*κ*B pathways were evaluated. Briefly, cells were exposed to TNF-*α* (100 ng/mL) for 15 min with or without pretreatment with DE (100 *μ*g/mL) for 24 h. With respect to TNF-*α*-induced MAPK activation, DE significantly inhibited the TNF-*α*-induced phosphorylation of JNK (Figure 2(b)). Furthermore, DE blocked the NF-*κ*B pathway, which was confirmed by analyzing nuclear protein levels of NF-*κ*B p65 (Figure 2(c)). Following TNF-*α* treatment on HUVECs, levels of cytosolic NF-*κ*B were decreased, whereas increased nuclear expression was noted. However, DE apparently inhibited the increase of NF-*κ*B p65 at nucleus with minimal change of cytosolic expression. In brief, DE inhibited TNF-*α*-activated MAPK and NF-*κ*B signaling pathways in endothelial cells, which are known markers involved in the expression of VCAM-1. In addition, DE increased the expression of HO-1 in HUVECs, similar to VSMCs (Figure 2(d)). 

### 3.4. Danshen Extract Inhibits Balloon Injury-Induced Neointimal Hyperplasia

**Figure 3:**
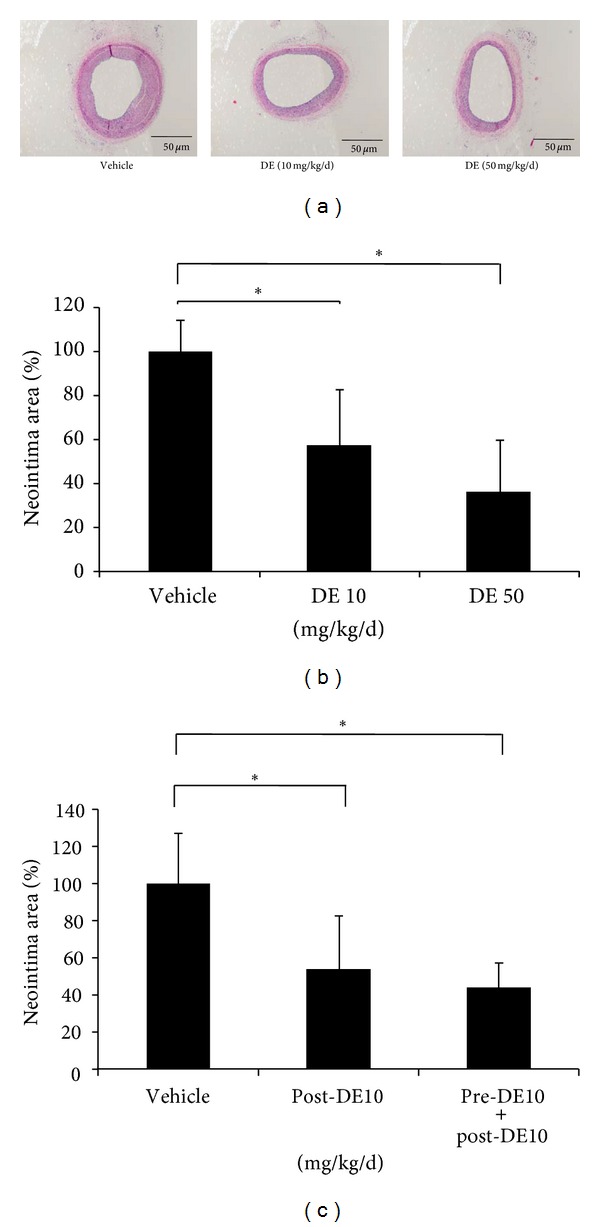
Effect of Danshen extract on neointimal formation after carotid artery balloon injury in rats. (a)-(b) Rats were orally administered one of two different concentrations of Danshen extract (DE) (10 and 50 mg/kg/d, both *n* = 7; represented as DE 10 and DE 50, resp.,) or vehicle (*n* = 7) every day from 2 weeks before and to four weeks after balloon injury. (a) Representative histologic sections of neointimal formation. Left, vehicle treatment; Middle, DE (10 mg/kg/d) treatment; Right, DE (50 mg/kg/d) treatment. (b) Neointimal formation after balloon injury according to the doses of treated DE. (c) Neointimal formation after balloon injury according to the existence of DE pretreatment. To evaluate the effect of DE pretreatment on neointimal hyperplasia, rats were subjected to DE treatment (10 mg/kg/d) for one of two periods of time: four weeks after injury, *n* = 9 (Post-DE 10); two weeks before injury until 4 weeks after injury, *n* = 9 (Pre-DE10 + Post-DE10). The neointimal formation was calculated by [intima/(media + intima)] × 100 (%). The bar indicates the standard error. *P < 0.05 for the indicated comparisons. DE: Danshen extract.

Balloon injury-stimulated neointimal hyperplasia in the carotid artery of SD rats was explicitly compared to sham-operated rats. With respect to the dose of DE, the observed effects were significantly prevented by both doses of DE treatment, compared to the normal saline-treated group (58% and 37% in the DE 10 mg/kg and DE 50 mg/kg treated groups, respectively, both *P* < 0.001) (Figures 3(a) and 3(b)). In aspect of the significance in DE pretreatment, both methods of DE treatment decreased neointimal hyperplasia to 42%, compared to the vehicle-treated group (55% and 42% in the DE 10 mg/kg only after the procedure, and DE 10 mg/kg both before and after procedure-treated groups, respectively, both *P* < 0.001) (Figure 3(c)). Although there was not statistically significance, pretreatment with DE showed a more intense inhibitory effect on neointimal hyperplasia compared to the DE treatment group without pretreatment after the procedure (Figure 3(c)). 

### 3.5. Danshen Extract Serves As a Blood Thinner *In Vivo* and *Ex Vivo*

**Figure 4:**
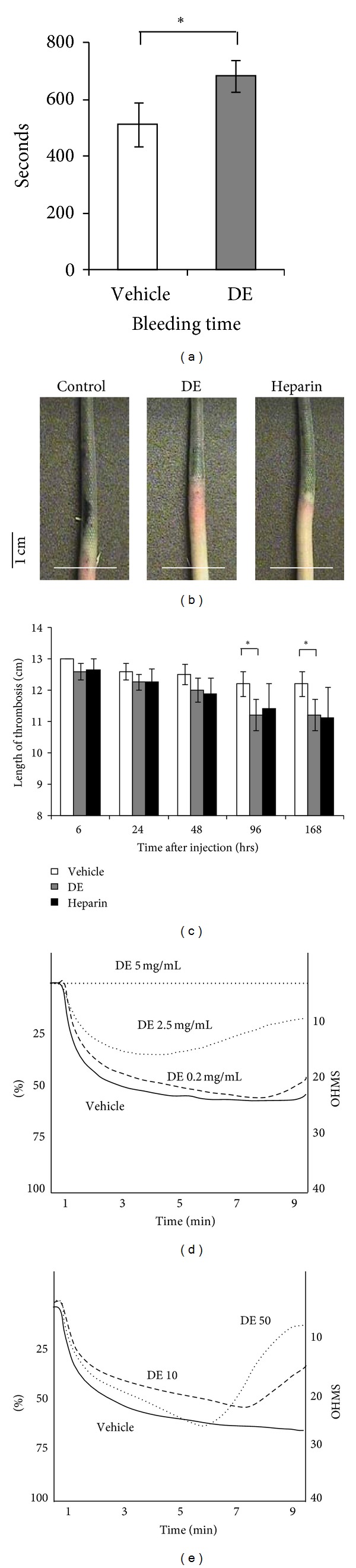
Blood thinning effect of Danshen extract. (a)–(c) Danshen extract (DE) pretreated at a dose of 10 mg/kg daily for 2 weeks via oral sonde (*n* = 6 in each group). (a) Bleeding time in response to treatment with DE. (b) Gross changes in the tail vein 96 h after *κ*-carrageenan injection. The white bar indicates the position 13 cm from the tail tip. The black bar indicates the reference length. (c) The length of thrombosis after *κ*-carrageenan injection. In the tail vein thrombosis rat model, DE treatment significantly inhibited thrombosis formation at 96 h after *κ*-carrageenan injection. (d) Platelet aggregation after DE treatment in *ex vivo* condition. The impedance aggregometer revealed that DE prevented platelet aggregation in a dose-dependent manner *ex vivo*. (e) Platelet aggregation after DE treatment in *in vivo* condition. Whole blood was sampled after DE pretreatment. Danshen extract (DE) administered at a dose of 10 mg/kg/d (DE 10) or 50 mg/kg/d (DE 50) for 2 weeks via oral sonde (*n* = 4 in each group). The platelet aggregation that was induced by ADP recovered more quickly with increasing doses of DE used in pretreatment. Graph of impedance aggregometer represented the mean value of each group. Results are expressed as the mean ± standard error. *P < 0.05 versus vehicle treatment. DE: Danshen extract.

DE treatment for two weeks resulted in a significantly prolonged bleeding time (*P* < 0.05) (Figure 4(a)). For induction of thrombosis, Beckmeier's modified model was utilized, specifically. Rats pretreated with or without DE were observed over a period of seven days after *κ*-carrageenan injection, analyzing tail length and color. During the first 2 to 3 h after *κ*-carrageenan injection, the tail swelled and turned red. Next, the tail changed to an auburn color around 6 h after *κ*-carrageenan injection. Finally, 96 h after *κ*-carrageenan injection, the injured tails of the DE pretreatment group had recovered significantly compared to the vehicle pretreatment group (*P* < 0.05). With respect to the formation of tail vein thrombosis, DE pretreatment produced the similar effect as heparin pretreatment (Figures 4(b) and 4(c)).

To evaluate the effect of DE on platelet activation, DE was administered using both *in vivo* and *ex vivo* models. In both models, DE inhibited the activation of platelets in a dose-dependent manner (Figures 4(d) and 4(e)). Interestingly, according to the i*n vivo* study results, DE treatment induced early recovery from platelet aggregation, which was confirmed with an impedance aggregometer (Figure 4(e)).

### 3.6. Danshen Extract Prevented In-Stent Restenosis

**Figure 5:**
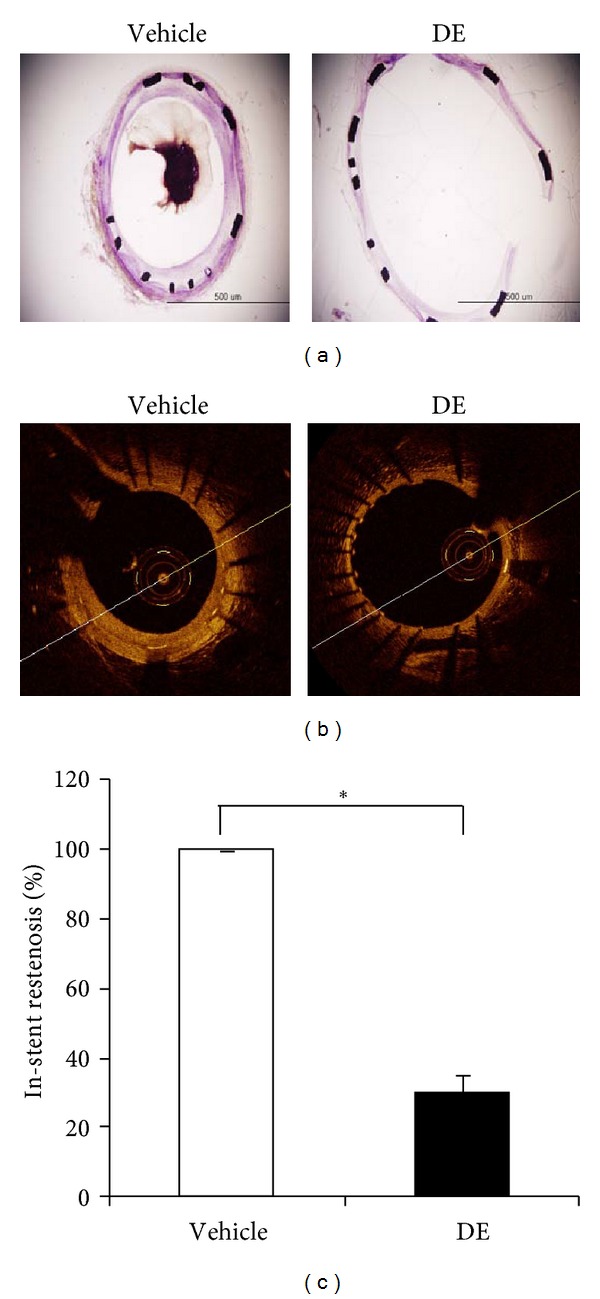
Effects of Danshen extract on in-stent restenosis. Rabbits were subcutaneously administered Danshen extract (DE; 20 mg/kg) twice a day from two weeks before to four weeks after the stent insertion. (a) Representative cross sections of stented iliac arteries in DE-treated and vehicle-treated rabbits 4 weeks after stent insertion. Sections were stained with hematoxylin and eosin; scale bar: 500 *μ*m. (b) Intravascular imaging for each iliac artery via intravascular optical coherence tomography (OCT). DE-treated rabbits showed significantly decreased in-stent restenosis. (c) Imaging analysis of in-stent restenosis, obtained from OCT. Results are expressed as the mean ± standard error. *P < 0.05 for the indicated comparisons. DE: Danshen extract.

Four weeks after stent insertion, the extent of in-stent restenosis was assessed in histological sections of stented rabbit iliac arteries. Compared with the levels in the control group treated with aspirin during procedure, DE treatment combined with aspirin markedly attenuated the formation of in-stent restenosis (Figure 5(a)). Furthermore, OCT technology demonstrated that the area of restenosis in the DE-treated group was decreased to 30% compared with the control group (Figures 5(b) and 5(c)). The size of restenosis was analyzed at six points in each stented artery. In addition, minimal numbers of both red and white thrombi were found in the DE-treated group, whereas these cells were ubiquitously present in control cells.

## 4. Discussion

Atherosclerosis is a multifactorial disease associated with various risk factors. The cellular pathogenesis of atherosclerosis is characterized by endothelial dysfunction, VSMC proliferation, and platelet aggregation. Importantly, these three factors play critical roles in the development of atherosclerosis and in the progress of cardiovascular disease [18, 19]. The mechanisms of aspirin and clopidogrel, which are two common medicines used in the treatment of atherosclerotic cardiovascular diseases, have various pathways affecting the vascular endothelium, VSMC, and platelets. However, these drugs have the potential for serious adverse reactions including hemorrhage and hematologic adverse reactions [20]. Furthermore, issues regarding resistance to aspirin and clopidogrel have led to the need for the development of newer agents. 

Recently, several studies have reported that natural products such as herbal medicines have therapeutic effects on cardiovascular diseases. However, these treatments face limited acceptance by the clinical opinion makers due to the lack of defined mechanisms involving their antiatherosclerotic effects. In this study we used three different animal models after analyzing the effects and mechanisms of DE *in vitro* with endothelial cells, VSMCs, and platelets. 

The proliferation and migration of VSMCs to the intima leads to neointimal hyperplasia and this process is triggered by multiple factors. Cytokines and growth factors such as PDGF, which is produced by platelets, VSMCs, and endothelial cells in the injured vascular wall involved in this process. Abnormal proliferation of VSMCs is thought to play an important role in the pathogenesis of atherosclerosis and restenosis after stent insertion. Recent evidence indicates that increased intracellular level of ROS is one of the main mechanisms in proliferation and migration of VSMCs [21, 22]. MnSOD and HO-1 have the roles in the atherogenic effect of ROS. MnSOD is located in the mitochondrial matrix and protects the mitochondria against oxidative stress [23]. Together with MnSOD, HO-1 expression also attenuates PDGF-induced VSMC proliferation and ROS production [24]. In this study, treatment with DE led to decreased ROS production, followed by an increased abundance of MnSOD and HO-1 proteins. In aspect of VSMCs migration, PI3K-Akt pathway is important to activate the NADH oxidase, leading to ROS production. It is well known that PI3K/Akt is the main PDGF signaling pathway and that this pathway is linked to numerous cellular processes, including both proliferation and migration [25]. DE inhibited phosphorylation of Akt, resulting in the accompanying inhibition of VSMC migration. Furthermore, wound-induced migration was significantly prevented by DE. Briefly, DE inhibited proliferation and migration, decreased the production of ROS, and inhibited the PI3K-Akt pathway in VSMCs.

TNF-*α*, one of the major inflammatory cytokines, mediates systemic inflammation and immune responses by enhancing the expression of adhesion molecules and the secretion of inflammatory mediators in the vascular endothelium [26]. Accumulating evidence suggests that the induction of VCAM-1 or ICAM-1 by TNF-*α* in endothelium is mediated by the activation of MAPK and transcription factors such as NF-*κ*B [13], leading to the pathogenesis of atherosclerosis. Although TNF-*α* alone significantly increased the level of VCAM-1 expression, DE pretreatment clearly inhibited VCAM-1 expression in HUVECs. In the present study, the change of VCAM-1 expression was linked to effect of TNF-*α* in HUVECs as well as balloon-injured endothelium of rats after DE treatment. With respect to the upstream molecules of VACM-1, MAPKs, which are a group of highly conserved serine/threonine kinases including JNK, participate in the vessel inflammatory reactions via integration of stimuli acting on cells, production of inflammatory mediators via the phosphorylation of downstream kinases, and the activation of transcription factors [27]. DE significantly inhibited TNF-*α*-induced phosphorylation of JNK in HUVECs. In this study, cytosolic NF-*κ*B was decreased following increased expression of NF-*κ*B protein in the nucleus after TNF-*α* treatment. However, treatment with DE apparently inhibited the NF-*κ*B pathway by preventing translocation. Thus, our data suggested that DE inhibited TNF-*α*-induced activation of the JNK and NF-*κ*B signaling pathways, which might contribute to the suppression of VCAM-1 in the endothelium.

Together with VSMCs and endothelial cells, thrombosis formation induced by platelets plays a major role in the development of atherosclerosis. In this study, we examined the antiplatelet and antithrombotic effects of DE. Platelet aggregation is a complex process that is mediated primarily through platelet adhesion at the injured site with the activation of endogenous agonists such as ADP, collagenase, and thrombin. These agonists stimulate platelet aggregation, resulting in thrombus formation through receptors on the platelet membrane [28]. In commercially popular agents, aspirin targets for the ADP. In this study, DE inhibited the ADP-induced aggregation of platelets in both *in vitro* and *in vivo* conditions. In the *in vivo* study with the impedance aggregometer, DE blocked the secondary activation of platelet aggregation in a manner similar to the effects of aspirin. Further study using multiple agonists for platelet activation could be helpful in understanding the antiplatelet effects of DE. The effect of DE on thrombus formation was studied using the rat model of tail vein thrombosis, a model that can be used to evaluate the effects of antithrombotic and thrombolytic agents [29]. Heparin treatment, which was used as a positive control agent, had similar effects on tail vein thrombosis as reported previously [29]. The antithrombotic effects of DE were similar to those of heparin. Furthermore, antithrombotic effect of DE was reconfirmed in rabbit iliac artery stent model. 

With results confirmed in both *in vitro* and *in vivo* rat models, the antiatherogenic effects of DE were analyzed in a rabbit animal model. The in-stent restenosis animal model is important for understanding thrombosis formation and vascular biology [30]. As seen with the rat models, DE had dramatic effects on the prevention of instent restenosis including vascular proliferation and thrombus formation in a rabbit model. 

The progression of atherosclerosis consists of multiple processes including, oxidative stress, migration and proliferation of VSMC, inflammation and dysfunction of endothelial cells, and thrombosis formation. In this study, DE showed multiple therapeutic functions on each pathophysiology of atherosclerosis. Multifunctional roles of DE on pathogenesis of atherosclerosis could be explained by the multiple components constituting the extracts. The need for development of natural compounds as therapeutics might be explained with this reason. However, further studies should be advanced to evaluate the key mechanism of DE on atherosclerosis. The key mechanism could present the specified indication as therapeutics.

Several studies demonstrated the effect of each DE component on atherosclerosis, suggesting the possible mechanisms. Until now, most studies evaluating the antiatherosclerotic effects of Danshen used lipid-soluble components and focused on it. In terms of the extract's water-soluble components, rosmarinic acid, lithospermic acid, ursolic acid, and salvianolic acid B have been reported to have antiatherogenic or antithrombotic effects [31–33]. Furthermore, we reported that protocatechuic aldehyde which is one of the main water-soluble components dissolved in DE inhibited migration and proliferation of VSMCs and intravascular thrombosis [34]. In aspect of molecular mechanisms, most of them had inhibitory actions on MMP-2/9, VACM-1, ICAM-1, and NF-*κ*B. However, the protective action of the each component had been achieved with the relative high concentration compared with DE used in this study. DE used in this study was not comprised of a single component but rather a mixture of the components of the extracts. This included mostly water-soluble agents because of the method of extraction. DE used in this study showed more stronger effects compared with previously reported results for either single components or combinations of dual components at similar molecular concentrations [35]. This means that DE in this study had synergistic effects or at least additional effects on preventing atherosclerosis compared with that from each single component of danshen. The higher effects with relative lower concentration of each component are important to make it useful in developing new therapeutics. The results of this study suggest not only the medical usefulness of DE but also the need for evaluation of underlying mechanism on interaction between each component. 

In conclusion, we have demonstrated that DE, with its prominent water-soluble components, exerts a protective effect against atherosclerosis by inhibiting multiple pathways associated with VSMCs, endothelial cells, and platelets. The antiatherogenic effects of DE were confirmed both *in vitro* and *in vivo*, including in rat and rabbit models. These results support the possibility of implementing water soluble DE as a therapeutic agent in the treatment of cardiovascular diseases. Further studies on the individual water-soluble components of DE are needed in order to evaluate the therapeutic effects of DE on cardiovascular diseases.

## Figures and Tables

**Table 1:** Ultra performance liquid chromatography—ultraviolet assay of Danshen extracts.

No.	Reference compound	Amount in DE 1 mg (*μ*g/mg)	Solubility
1	Salvianolic acid A (danshensuan A)	22.959	Water
2	Dihydroxybenzoic acid (Protocatechuic acid)	0.41	Water
3	Dihydroxybenzaldehyde (Protocatechuic aldehyde)	19.37	Water
4	Caffeic acid	4.34	Water
5	Rosmarinic acid	87.558	Water
6	Lithospermic acid	30.298	Water
7	Salvianolic acid B	133.932	Water
8	Salvianolic acid A	53.763	Water
9	Dihydrotanshinone I	<0.205	Lipid
10	Cryptotanshinone	<0.273	Lipid
11	Tanshinone I	<0.236	Lipid
12	Tanshinone IIA	<0.661	Lipid
